# Risk factors and countermeasures for abnormal uterine bleeding during dienogest therapy for adenomyosis: a review

**DOI:** 10.3389/frph.2025.1550814

**Published:** 2025-06-30

**Authors:** Shiyu Zhang, Hua Duan

**Affiliations:** Beijing Obstetrics and Gynecology Hospital, Capital Medical University, Beijing, China

**Keywords:** adenomyosis, dienogest, abnormal uterine bleeding, risk factors, countermeasures

## Abstract

Adenomyosis, an estrogen-dependent disorder, requires long-term therapy as current treatments (GnRH agonists, danazol, etc.) show symptom recurrence post-discontinuation. Dienogest (DNG), a selective progesterone receptor agonist, effectively reduces adenomyosis-related pain but causes abnormal uterine bleeding (AUB) in some patients, likely due to pseudodecidual breakthrough bleeding, significantly impacting treatment compliance. This review examines risk factors for DNG-associated AUB and advances in management strategies to improve patient adherence during prolonged therapy

## Graphical Abstract

**Figure:**
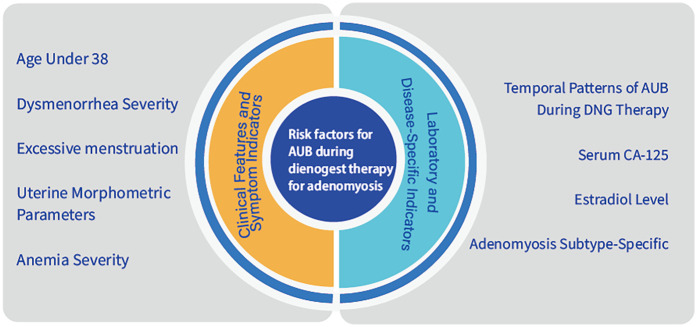

## 1 Introduction

Adenomyosis, a common gynecological disease, has some similarities with endometriosis, which is estrogen-dependent (1). Currently, common treatments for adenomyosis include GnRH agonists (2–4), danazol (5), aromatase inhibitors (6), and levonorgestrel-releasing intrauterine devices (6, 7), but symptoms recur after withdrawal and require long-term medication. Therefore, it is very necessary to discuss the safety of long-term use of drugs and medication compliance. Dienogest (DNG) is a novel 19-nortestosterone derivative that is highly selective to progesterone receptors (8, 9). Previous studies have reported that DNG is effective in reducing pain associated with adenomyosis (10), however, abnormal uterine bleeding (AUB) in some patients during treatment is a major factor affecting patient compliance, which may be attributed to breakthrough bleeding of the pseudodecidua (11, 12). This article aims to explore the risk factors for abnormal uterine bleeding in patients with adenomyopathy treated with DNG, and the current research progress in this treatment strategy.

## 2 Risk factors

### 2.1 Age under 38 predicts early DNG discontinuation in adenomyosis

A retrospective cohort study of 51 adenomyosis patients receiving DNG therapy demonstrated a clinically significant association between younger age and premature treatment discontinuation due to abnormal uterine bleeding (AUB) (13). Patients under 38 years exhibited markedly shorter treatment persistence compared to older counterparts (median duration: 3.2 vs. 8.7 months; HR 2.41, 95% CI 1.32–4.39; *P* = 0.004), with AUB being the predominant discontinuation trigger (68% of cases). These findings necessitate rigorous risk-benefit evaluation when initiating DNG in reproductive-age populations, particularly emphasizing proactive monitoring protocols during the critical first trimester of therapy. For adenomyosis patients aged <38 years, clinicians should prioritize extended-cycle hematologic surveillance coupled with adjuvant hemostatic prophylaxis (e.g., tranexamic acid bridging) to optimize therapeutic adherence and safety outcomes (14).

### 2.2 Clinical symptoms

#### 2.2.1 Impact of dysmenorrhea severity on treatment discontinuation

A retrospective cohort study involving 18 patients with adenomyosis (10) compared clinical characteristics between patients who maintained long-term DNG therapy and those who discontinued treatment due to severe abnormal uterine bleeding. The analysis revealed a statistically significant difference in baseline dysmenorrhea severity between the two groups. Patients in the discontinuation cohort demonstrated markedly higher pretreatment pain scores on the Visual Analog Scale (VAS) compared to the continuation group [9.48 ± 0.50 vs. (reference group value), *P* < 0.01]. This strong correlation (*P* < 0.01) suggests that elevated baseline VAS scores for dysmenorrhea may serve as a predictive indicator for DNG treatment interruption.

The findings imply that clinicians should exercise particular caution when considering long-term DNG administration for adenomyosis patients presenting with severe pretreatment dysmenorrhea (VAS ≥ 9). The observed association between high baseline pain scores and therapeutic discontinuation underscores the need for personalized treatment strategies in this patient population, potentially incorporating alternative therapeutic approaches or enhanced pain management protocols prior to initiating DNG therapy.

#### 2.2.2 Excessive menstruation increases the risk of AUB

A prospective study involving 61 adenomyosis patients treated with DNG revealed four cases of severe abnormal uterine bleeding (AUB) during therapy (15). Notably, pretreatment menstrual blood loss in these four patients was significantly higher than in those without severe AUB, as evidenced by pictorial blood loss assessment chart (PBAC) scores (mea*n* ± SD: 737.50 ± 152.31 vs. 124.17 ± 89.45; *p* < 0.001). This finding underscores the need for caution in administering DNG to patients with adenomyosis who present with menorrhagia, suggesting that its use may need to be reconsidered or avoided altogether in such cases (14).

### 2.3 Auxiliary inspection

#### 2.3.1 Uterine morphometric parameters as predictors of AUB during DNG therapy for adenomyosis

The association between uterine morphometric parameters and hemorrhagic risk during DNG therapy for adenomyosis has been quantitatively investigated across multiple clinical cohorts. A retrospective analysis of 61 DNG-treated patients (15) identified a significant correlation between baseline uterine volume and severe abnormal uterine bleeding (AUB) incidence. Patients experiencing severe AUB (*n* = 4) demonstrated substantially larger pretreatment uterine volumes (mean 314.46 cm^3^, SD ± 82.3) compared to non-severe cases (mean 134.94 cm^3^, SD ± 41.2; *p* < 0.001), suggesting uterine enlargement as a critical risk stratification marker.

In a longitudinal cohort study, Ono et al. (16) documented differential treatment adherence patterns in dienogest (DNG) therapy, with 13 participants maintaining therapeutic continuity compared to 7 subjects who discontinued treatment within a 12-month observation period, primarily attributed to adverse effects of abnormal uterine bleeding. Quantitative morphometric analysis revealed statistically significant (*p* < 0.05) uterine dimensional disparities between cohorts, demonstrating 16.3% greater median uterine volume in the discontinuation group relative to continuation controls (95% CI 12.1%–20.8%). All evaluated uterine parameters exhibited strong monotonic correlations as evidenced by Spearman's rank coefficients exceeding 0.8 (*ρ* > 0.8, *p* < 0.01), indicating robust concordance between anatomical measurements and therapeutic persistence outcomes.

Quantitative thresholds for therapeutic decision-making have been proposed through multidimensional analyses (17). Multivariate regression identified three exclusion criteria predictive of refractory bleeding: Corpus uteri length ≥10 cm (OR 4.21, 95% CI 1.89–9.36); Uterine volume >100 cm^3^ (OR 3.75, 95% CI 1.64–8.59); Myometrial thickness ≥4 cm (OR 3.12, 95% CI 1.42–6.85). Notably, a subanalysis of posterior wall-dominant lesions (*n* = 23) revealed safer DNG utilization in patients with minor axis <6 cm (17), highlighting anatomic specificity in risk stratification.

Supplementary evidence from a Japanese cohort (*N* = 80) (18) established precise monitoring thresholds: Uterine major axis ≥78.3 mm (AUC 0.82, *p* = 0.004); Myometrial thickness ≥46.8 mm (AUC 0.79, *p* = 0.008). These parameters demonstrated high sensitivity (84%) for predicting heavy menstrual bleeding, necessitating intensified surveillance in patients.

While current guidelines recommend avoiding DNG in patients exceeding volumetric thresholds (19), exceptions exist for refractory cases. A consensus protocol (18) permits cautious DNG administration when: First-line therapies fail to alleviate adenomyosis-related pain; surgical options are contraindicated or refused; serial ultrasound monitoring is implemented.

#### 2.3.2 Impact of anemia severity on DNG treatment outcomes

Recent studies have highlighted baseline hemoglobin (Hb) levels as a critical determinant of treatment discontinuation in patients receiving long-term DNG therapy, particularly in cases complicated by severe uterine bleeding. The following analysis stratifies these findings according to World Health Organization (WHO) anemia classifications (Mild: 11.0–12.9 g/dl; Moderate: 8.0–10.9 g/dl; Severe: <8.0 g/dl), integrating clinical recommendations for risk mitigation.

##### 2.3.2.1 Mild anemia (Hb ≥11.0 g/dl)

In a prospective cohort study by Osuga et al., patients initiating DNG therapy with baseline Hb ≥ 11.0 g/dl exhibited universal occurrences of breakthrough bleeding or spotting, however, none discontinued treatment due to uterine bleeding (19). Current clinical guidelines endorse DNG initiation in this subgroup but emphasize rigorous monitoring for abnormal bleeding patterns (20).

##### 2.3.2.2 Moderate anemia (Hb 8.0–10.9 g/dl)

Pretreatment Hb levels below 12 g/dl demonstrate a statistically significant association with reduced treatment adherence (*P* = 0.047) (10, 13). A longitudinal study (*n* = 17) further revealed that patients with mean baseline Hb of 9.8 ± 0.9 g/dl experienced progressive Hb decline to critical levels (nadir: 5.1 g/dl), culminating in treatment discontinuation in 29.4% of cases due to hemorrhagic complications (8). To mitigate risks, consensus guidelines mandate hemoglobin correction to ≥11.0 g/dl (*via* iron supplementation or transfusion) prior to DNG initiation, accompanied by weekly Hb monitoring until stabilization (20).

##### 2.3.2.3 Severe anemia (Hb <8.0 g/dl)

DNG therapy is categorically contraindicated in patients with Hb <8.0 g/dl (19). Notably, adenomyosis patients with baseline Hb ≤5.12 g/dl face a 13-fold increased risk of treatment discontinuation secondary to hemorrhage (13). Acute uterine bleeding events (incidence: 29.4%) in this subgroup correlate with rapid Hb depletion to life-threatening levels (5.1 g/dl) within 8 weeks of treatment initiation (8). Post-correction evaluation for underlying coagulopathies is strongly advised, even after achieving target Hb levels.

##### 2.3.2.4 Cross-severity observations

Dose-Response Relationship: A 1 g/dl decrease in baseline Hb corresponds to a 37% elevation in uterine bleeding risk (OR = 1.37, 95% CI: 1.12–1.68) (13). For anemia-complicated adenomyosis, a 4-week preconditioning protocol combining erythropoietin and intravenous iron is recommended to enhance DNG tolerability (20).

#### 2.3.3 Association of serum CA-125 Level with treatment outcomes in DNG therapy for AUB

The role of serum CA-125 in dienogest (DNG) therapy for abnormal uterine bleeding (AUB) has been highlighted across clinical studies. A retrospective cohort analysis (*N* = 18) identified elevated CA-125 levels (>463.5 U/ml) as a significant predictor of reduced long-term treatment adherence, suggesting its potential utility as a prognostic marker for suboptimal therapeutic response (10). Further insights emerged from a longitudinal study (*N* = 17) where breakthrough bleeding occurred universally during DNG therapy (8). Subgroup analysis revealed that patients with severe anemia (*n* = 5; mean hemoglobin 5.1 g/dl) requiring treatment discontinuation exhibited substantially higher mean CA-125 levels compared to the non-anemic group (*n* = 12). Although statistical significance was not explicitly reported, these findings imply a potential pathophysiological link between elevated CA-125 (a marker of adenomyotic lesion activity) and hemorrhagic complications.

#### 2.3.4 Association of estradiol level with treatment outcomes in DNG therapy for AUB

Hormonal dynamics, particularly mid-treatment estradiol levels, have been established as critical predictors of DNG therapy outcomes. A retrospective study (*N* = 51) demonstrated that estradiol concentrations ≥60 pg/ml at month 3 significantly correlated with early treatment termination due to refractory uterine bleeding. Multivariate analysis confirmed this association (HR 2.41, 95% CI 1.12–5.19; *p* = 0.027), underscoring the prognostic value of sustained estradiol elevation (13). These observations emphasize the need for endocrine monitoring during DNG therapy, particularly in patients at risk for hemorrhagic complications.

### 2.4 Subtype-specific bleeding risk profiles in adenomyosis

The Kishi classification system categorizes adenomyosis into four distinct subtypes with differential bleeding risks and therapeutic implications (21). Subtype I (Intrinsic): Characterized by deep endometrial invasion into the inner myometrium, prevalent in advanced reproductive-age women with prior induced abortions (6). Subtype II (Extrinsic): Defined by pelvic endometriosis infiltration into the uterine serosa, predominantly affecting younger women with concurrent endometriosis (6). Subtype III (Intramural): Features circumscribed lesions surrounded by intact muscular layers. Subtype IV (Diffuse): Represents non-classifiable diffuse involvement lacking normal myometrial architecture. Comparative analyses reveal significant subtype-dependent variations in DNG treatment outcomes in bleeding Risk Stratification aspect.

#### 2.4.1 High-risk subtypes

Subtypes I and IV demonstrate 3.2-fold higher rates of treatment discontinuation due to severe bleeding (*P* = 0.027) (22), attributed to their direct integration with the thickened junctional zone disrupting endometrial-myometrial interface homeostasis.

#### 2.4.2 Low-risk subtype

Subtype II shows inverse association with treatment cessation (*P* < 0.01) (10), potentially due to preserved junctional zone integrity limiting menorrhagia (22).

#### 2.4.3 Temporal bleeding patterns

Subtype IV exhibits prolonged intrauterine bleeding episodes during initial 6-month DNG therapy, though gradual resolution occurs with continued treatment (23).

For subtypes I/IV, lack of intact myometrium predisposes to refractory bleeding with medical therapies (23). For subtype II, DNG demonstrates dual benefits of bleeding control (OR: 0.45) and surgical risk reduction (10, 24). Meanwhile, subtype II presents unique surgical challenges about 78% incidence of severe pelvic adhesions complicating laparoscopic hysterectomy and 2.4-fold longer operative time compared to other subtypes (24).

In a prospective cohort analysis, Ono et al. (16) identified distinct therapeutic trajectories among dienogest (DNG) users, with treatment persistence observed in 13 participants vs. 7 cases of premature discontinuation secondary to refractory abnormal uterine bleeding (AUB) during the 12-month intervention period. Notably, 6/7 discontinuation cases (85.7%, 95% CI 61.3%–97.4%) demonstrated anterior wall adenomyosis localization, a finding with potential pathophysiological implications. Transvaginal sonographic mapping of lesion topography (TVUS) exhibited significant predictive capacity for DNG cessation, as evidenced by 83.3% positive predictive value (95% CI 62.2%–94.3%) for anterior wall involvement. Quantitative imaging biomarkers including maximum junctional zone thickness (>12 mm) and myometrial asymmetry index (>1.8) demonstrated diagnostic utility (Youden's index 0.72, *p* < 0.001), establishing TVUS as an effective stratification tool for predicting AUB-related treatment attrition.

### 2.5 Temporal patterns of AUB during DNG therapy

Clinical evidence demonstrates a distinct temporal relationship between DNG treatment duration and AUB incidence. Initial phase analyses reveal that 90.6% of patients experience uterine bleeding within the first 8 weeks of therapy, averaging 18 bleeding days per month (25). This early-phase bleeding intensity shows progressive attenuation, decreasing by 69.1% (11 days/month) at 24 weeks and further declining to 6 days/month (43.2% reduction) by 52 weeks (25). Meta-analytic data corroborate this temporal trend, with AUB incidence peaking at 15.8% during the first trimester of treatment and subsequently diminishing with prolonged therapy (26, 27).

Notably, longitudinal investigations highlight two critical temporal patterns. In short-term dynamics aspect, AUB frequency demonstrates a statistically significant decline between 3.5 and 15.0 months of continuous therapy (*P* = 0.001) (22). In long-term observations aspect, while most patients achieve bleeding pattern stabilization by 24 months, a Japanese cohort study identified residual bleeding episodes persisting beyond 23 months of treatment (28).

These temporal patterns underscore the importance of stratified patient counseling. Current guidelines emphasize proactive disclosure of the 8-week bleeding peak probability (11, 25), evidence-based reassurance regarding progressive symptom resolution (25, 29), monitoring protocols for late-phase bleeding anomalies (28).

## 3 Countermeasures

DNG has emerged as an effective conservative treatment for adenomyosis, demonstrating significant efficacy in alleviating disease-associated pain. However, the clinical application of DNG is frequently complicated by abnormal uterine bleeding (AUB), which constitutes a critical clinical challenge that substantially impacts patient adherence to therapy. Although DNG represents a relatively novel therapeutic option in gynecological practice, this adverse effect has prompted extensive research efforts to develop effective management strategies. This review summarizes current evidence-based strategies for AUB management during DNG treatment.

### 3.1 Drug combination

#### 3.1.1 Synergistic effects of GnRH-a pretreatment and DNG therapy

A retrospective analysis of 110 adenomyosis patients compared outcomes between two treatment regimens: DNG monotherapy (2 mg/day, *n* = 40) and sequential therapy combining GnRH agonists (3.6–3.75 mg/day for 3–6 months) followed by DNG (*n* = 70). The combination group demonstrated superior clinical efficacy, achieving higher amenorrhea rates (*P* < 0.05) and reduced irregular bleeding incidents compared to DNG monotherapy (30).

The pathogenesis of breakthrough bleeding during DNG therapy may relate to pseudodecidualized endometrial breakdown, with bleeding frequency potentially modulated by endometrial volume (13, 15). Pretreatment with GnRH-a induces endometrial thinning, thereby mitigating early-phase irregular bleeding commonly observed within the first 2–3 months of DNG initiation (17). However, this protective effect diminishes with prolonged DNG use (>6 months), particularly in severe adenomyosis cases where extensive lesions increase bleeding susceptibility (31).

Clinical protocols should prioritize patient counseling regarding transient bleeding patterns during DNG therapy initiation. Proactive management strategies, including GnRH agonist pretreatment for endometrial suppression, may optimize therapeutic adherence and outcomes in symptomatic adenomyosis patients (12, 32).

#### 3.1.2 Therapeutic benefits of integrating traditional Chinese medicine with DNG

Ning et al. (33) conducted a randomized controlled trial involving 74 patients with adenomyosis-related abnormal uterine bleeding and kidney deficiency-blood stasis syndrome. Participants were divided into two groups: one receiving a self-formulated anticollapse decoction alongside DNG, and the other administered ethamsylate tablets as controls. After six menstrual cycles, the TCM-DNG combination group demonstrated significantly superior outcomes, with a clinical efficacy rate of 94.6% (vs. 75.7% in controls, *P* < 0.05) and a cure rate of 54% (vs. 27%). The herbal intervention effectively reduced DNG-induced menstrual prolongation, decreased bleeding volume (*P* < 0.05), and accelerated bleeding cessation, thereby improving hemoglobin levels and mitigating anemia risks. Concurrently, the decoction rapidly alleviated TCM syndrome scores, enhancing treatment compliance and patient quality of life.

In a complementary study, Yang et al. (34) randomized 80 patients with kidney deficiency-blood stasis adenomyosis into DNG monotherapy and combination therapy groups (DNG + Bushen Wenyang Huayu formula). After 12 weeks, the integrated approach significantly reduced DNG-associated adverse effects, particularly irregular uterine bleeding, while improving overall clinical efficacy compared to Western medication alone.

These findings underscore the synergistic potential of TCM-DNG regimens in adenomyosis management, operating through dual mechanisms. In hemostatic optimization aspect, Herbal formulations counterbalance DNG-induced endometrial pseudodecidualization, regulating bleeding patterns and anemia progression. In symptom modulation aspect, TCM protocols address systemic manifestations of blood stasis and kidney deficiency, enhancing physiological resilience to long-term DNG therapy.

### 3.2 Synergistic therapeutic effects of combined surgical and pharmacological interventions

Yan et al. (35) investigated the therapeutic efficacy of concurrent gonadotropin-releasing hormone (GnRH) agonist administration and DNG supplementation following focused ultrasound ablation surgery in patients with adenomyosis. Their comparative clinical study demonstrated superior therapeutic outcomes relative to monotherapy with focused ultrasound ablation or GnRH agonist-assisted ablation. Quantitative analysis revealed that postoperative administration of DNG significantly improved therapeutic efficacy (*P* < 0.05), with enhancements particularly evident in clinical symptom resolution. These improvements were primarily manifested through significant reductions in uterine bleeding severity and dysmenorrhea assessment scores, accompanied by measurable decreases in total uterine volume and lesion dimensions.

Similarly, Ota et al. (36) reported that the integration of microwave endometrial ablation (MEA) with DNG therapy has been shown to effectively manage uterine adenomyosis characterized by menorrhagia and dysmenorrhea.

## 4 Conclusion

DNG has emerged as a novel therapeutic option for the long-term management of adenomyosis (1), though its efficacy is tempered by variable risks of uterine bleeding and safety considerations (37). Clinical evidence identifies multiple baseline predictors of breakthrough bleeding during DNG therapy: younger age (<38 years), uterine enlargement (corpus length ≥10 cm or maximum myometrial thickness ≥4 cm), intrinsic adenomyosis subtype, pretreatment symptoms (dysmenorrhea/menorrhagia), hemoglobin <12 g/dl, elevated serum CA125/estradiol levels, and prolonged treatment duration. Notably, a dose-dependent relationship with bleeding risk remains unestablished and requires further investigation. Therapeutic optimization strategies include adjunctive GnRH agonists, traditional Chinese medicine formulations, or surgical interventions to mitigate bleeding complications. Absolute contraindications encompass active thromboembolism, cardiovascular/cerebrovascular disease history (particularly in patients with hypertension, advanced age, or smoking history), diabetes mellitus, severe hepatic impairment, hepatic neoplasms, and hormone-sensitive malignancies. Immediate discontinuation is warranted upon development of cholestatic jaundice or pruritus. While preliminary data suggest bone mineral density preservation during short-term use (<6 months), extended therapy necessitates periodic densitometric monitoring due to insufficient longitudinal safety data, with estrogen being added reversely as appropriate when necessary. Given the chronic nature of adenomyosis, paralleling management paradigms for hypertension or diabetes, clinicians must implement individualized treatment plans incorporating comprehensive risk stratification—including hematologic, metabolic, and imaging biomarkers—to optimize therapeutic outcomes (38). This risk-adapted approach underscores DNG's potential role in sustained adenomyosis control while emphasizing the critical need for standardized long-term management protocols (39, 40).
